# The Effects of Congelation upon Water

**Published:** 1864-03

**Authors:** 


THE EFFECTS OF CONGELATION UPON WATER.—Dr. Robinet, a member of the Academy of Medicine, Paris, has published an account of experiments conducted by him to test the effects of congelation upon drinking water. It is well known that the ice which is formed in the sea yields nothing but fresh water, all the salt having been eliminated by congelation. In the Northern parts of Europe this property is turned to account for the extraction of salt from sea water; for a large sheet of the latter having been left to freeze, the ice is afterwards cut away, and the unfrozen water left below is so rich in salt as to require very little evaporation to yield it in a solid state. This property will also serve to analyze wine. Suppose it was required to determine the quantity of water fraudulently added to a certain wine; by exposing it to the action of artificial refrigeration, all the water would be alone, and the wine left in its purity. By a similar process, ships at sea, being short of water, might be supplied with this necessary article. We will suppose the temperature of sea water under the tropics to be 300 centigrade. If a quantity be exposed in a vessel to the action of a mixture of sulphate of soda and hydrocloric acid, two very cheap commodities, the temperature of the water will fall to 100 below freezing point. Let it then be exposed to a second mixture of the same kind, generally eight parts of sulphate to five of the acid, and the temperature may be lowered to 170 below freezing point. Congealed water is then obtained free from salt, and may be used with impunity. Dr. Robinet has added a new fact to this theory by showing that the water of springs and rivers loses all its salts by congelation. These salts are chiefly those of lime and magnesia. The water subjected to experiment was that of the lakes of the Bois de Boulogne, the ice of which was found to be entirely free from the above mentioned salts. Such, indeed, is the chemical purity of the water thus obtained, that it may in most instances be substituted for distilled water.—*Scientific American.*

